# Notes on the Examination of the Sputum, Vomit, Fæces, Urine and Blood

**Published:** 1892-03

**Authors:** 


*Notes on the Examination of the Sputum, Vomit, Fæces, Urine and Blood.* By SIDNEY COUPLAND, M.D., F.R.C.P. Second Edition. Pp. 57. London: H. K. Lewis. 1891.

That a second edition of Dr. Coupland's *Notes* has been called for within nine months of the first issue shows that it supplies a real want. It will be found an useful aid to students in carrying out their clinical duties, and its value is increased in this edition by the addition of a chapter on the examination of the blood.

